# Deep neural network based tissue deconvolution of circulating tumor cell RNA

**DOI:** 10.1186/s12967-023-04663-w

**Published:** 2023-11-04

**Authors:** Fengyao Yan, Limin Jiang, Fei Ye, Jie Ping, Tetiana Y. Bowley, Scott A. Ness, Chung-I Li, Dario Marchetti, Jijun Tang, Yan Guo

**Affiliations:** 1 Department of Public Health and Sciences, Sylvester Comprehensive Cancer Center, University of Miami, Miami, FL 33136 USA; 2 Department of Computer Science, University of South Carolina, Columbia, SC 29208 USA; 3 Department of Biostatistics, Vanderbilt University Medical Center, Nashville, TN 37232 USA; 4 Department of Medicine, Vanderbilt University Medical Center, Nashville, TN 37232 USA; 5 Department of Internal Medicine, Comprehensive Cancer Center, University of New Mexico, Albuquerque, NM 87131 USA; 6 Department of Statistics, National Cheng Kung University, Tainan, 701401 Taiwan

## Abstract

Prior research has shown that the deconvolution of cell-free RNA can uncover the tissue origin. The conventional deconvolution approaches rely on constructing a reference tissue-specific gene panel, which cannot capture the inherent variation present in actual data. To address this, we have developed a novel method that utilizes a neural network framework to leverage the entire training dataset. Our approach involved training a model that incorporated 15 distinct tissue types. Through one semi-independent and two complete independent validations, including deconvolution using a semi in silico dataset, deconvolution with a custom normal tissue mixture RNA-seq data, and deconvolution of longitudinal circulating tumor cell RNA-seq (ctcRNA) data from a cancer patient with metastatic tumors, we demonstrate the efficacy and advantages of the deep-learning approach which were exerted by effectively capturing the inherent variability present in the dataset, thus leading to enhanced accuracy. Sensitivity analyses reveal that neural network models are less susceptible to the presence of missing data, making them more suitable for real-world applications. Moreover, by leveraging the concept of organotropism, we applied our approach to trace the migration of circulating tumor cell-derived RNA (ctcRNA) in a cancer patient with metastatic tumors, thereby highlighting the potential clinical significance of early detection of cancer metastasis.

**Supplementary Information:**

The online version contains supplementary material available at 10.1186/s12967-023-04663-w.

## Introduction

Cancer constitutes a prominent global cause of mortality, with significant implications for public health. According to the World Health Organization, cancer accounted for about 10 million deaths in 2020. Alarming projections indicate that by the year 2040, the number of new cancer diagnoses is estimated to escalate to 29.5 million, while the corresponding number of cancer-related deaths is expected to rise to 16.4 million. It is noteworthy, as highlighted by the American Cancer Society, that approximately 90% of cancer-related deaths can be attributed to metastasis, which denotes the dissemination of cancer cells from the primary tumor site to distant locations within the body. Steps of metastasis consist of detachment of the cells from the primary tumor into blood their transport through the blood stream, and dissemination to organ sites distant from the primary lesions and establishment of tumors in these sites. Once metastasis has occurred, the treatment of the disease becomes increasingly challenging. The early detection of metastasis bears significant clinical implications, as it allows for timely adjustments in treatment strategies [1].

The utilization of machine-learning techniques in cancer research has experienced a remarkable surge in recent years [2]. One notable application of such techniques involves the detection of tissue origin through the analysis of circulating genomic information gleaned from blood samples. For instance, Moss et al. established a model capable of predicting tissue origin based on methylation patterns of circulating cell-free DNA (cfDNA) [3]. They employed a methodology known as non-negative least squares linear regression (NNLS), which is commonly employed to predict continuous values by constraining all model parameters to be greater than or equal to zero. Subsequently, Larson et al. developed a distinct model using quadratic linear regression to predict the tissue origin of circulating cell-free RNAs (cfRNA) [4]. Although the regression methods utilized in these studies differed, they shared a common requirement: the utilization of a reference panel containing the average or median of the feature levels.

The reference-based approach has been extensively employed in the realm of biological research, notably exemplified by the widely recognized PAM50 method that employs a 50-gene panel to effectively classify breast cancer intrinsic subtypes [5]. Although the reference panel approach yields sufficiently accurate results, there exists a notable opportunity for substantial improvement. One of the key limitations associated with the reference panel approach pertains to the loss of information incurred during panel construction, necessitating the computation of average or median values across all samples and for all features. Consequently, this procedure results in the exclusion of feature-specific information pertaining to individual samples. In contrast, we propose the adoption of a neural network-based approach, wherein the model training process encompasses the consideration of feature information from all individuals within the training dataset. This alternative approach effectively captures the inherent variation present within the data, thus enhancing accuracy and providing a more comprehensive analysis framework.

In this study, we show that in addition to circulating cfDNA [3] and circulating cfRNA [4], tissue deconvolution can also work on circulating tumor cell RNA (ctcRNA) which are RNAs sloughed off the tumor cells which extravasate into circulating blood. Because circulating tumor cells (CTCs) are shed from tumors and may contribute to the establishment of metastatic lesions, their analysis, including ctcRNA, could potentially offer insights into disease progression and metastatic potential [6]. For example, other researchers demonstrated the feasibility of detecting and analyzing ctcRNA in patients with metastatic breast, prostate, and lung cancers [7]. The study showed that ctcRNA analysis could provide genetic information about the tumor and potentially guide treatment decisions. One phenomenon often associated with metastasis is organotropism which refers to the preferential or selective metastasis of tumor cells to specific organs in the body according to cancer types. Target organ’s metastatic specificity is characterized by the ability of certain tumor types to display a propensity for metastasizing to particular organs, often influenced by the interactions between tumor cells and the unique microenvironment of the target organ. Metastasized tumor cells can adapt to the local environment and interact with the surrounding cells/tissues often exhibiting characteristics of the local environment [8]. Building upon the concept of organotropism, our hypothesis centers upon the feasibility of tissue deconvolution using ctcRNA alongside cfDNA and cfRNA. We propose that ctcRNA-based tissue deconvolution holds considerable potential for clinical applications, particularly in the early detection of metastasis.

## Materials and methods

### Public RNA-seq data

Gene expression data for different tissue sites were obtained from the Genotype-Tissue Expression (GTEx) project (v8) [9] to serve as the discovery data for tissue-specific genes and training data for model development. Gene expression data from adjacent normal tissue and tumors were downloaded from The Cancer Genome Atlas to serve as validation data for gene-tissue specificity and model validation.

### Normal tissue RNA-seq

Normal tissues from six sites (brain, breast, colon, kidney, liver lung) were acquired from Human Tissue Repository, University of New Mexico. The tissues were processed by Analytical and Translational Genomics Shared Resource, University of New Mexico. Total RNA was extracted. Synthesis of cDNA and library preparation were performed using the SMARTer Universal Low Input RNA Kit for Sequencing (Takara) and the Ion Plus Fragment Library Kit (ThermoFisher). Each tissue type was uniquely barcoded. Sequencing was performed using the Ion Proton S5/XL systems (ThermoFisher). RNA-seq data was demultiplexed for further validation of our model. The normal tissue RNA-seq data was used as an independent validation dataset for our model (Additional file 1).

### Patient, ctcRNA isolation and sequencing

The patient was diagnosed with primary melanoma and enrolled to study according to protocols approved by the Institutional Review Board at UNM Health Sciences Center (UNM-HSC), Albuquerque, New Mexico (USA). The patient’s blood sample was collected after receiving informed written consent, according to the principles of Declaration of Helsinki. Peripheral blood (18 mL) was collected in CellSave™ (Menarini Silicon Biosystems, Inc.). Multiparametric flow cytometry was used to deplete Lin− and Lin+ cells from the peripheral blood (14.5–18 mL) of this melanoma brain-metastatic patient. The longitudinal monitoring of patient was performed by collecting blood and extracting RNA every 3 months over the period of 9 months. Unsupervised hierarchical clustering was performed to compare Lin− cell population and healthy donor blood. Blood collection was performed at the middle of vein puncture as part of patients’ routine clinical care. Following blood collection, the sample was immediately sent to the laboratory for isolation and analysis of CTCs. All blood specimens were analyzed within 24 h following blood draw.

To isolate peripheral blood mononuclear cells (PBMCs) from whole blood per our previous report [10], briefly, patient blood was lysed with red blood cell lysis buffer (BioLegend, Cat#420302, San Diego, CA, USA), and washed twice with 1× PBS (with 5 mmol/L EDTA from USB, Cat#15694, Cleveland, OH, USA). PBMCs were quantified by the countess II cell counter machine (Thermo Fisher, Waltham, MA, USA). Following cell blocking with Fc block (BioLegend, Cat# 422302, San Diego, CA, USA), PBMCs were stained for fluorescence labeling with FITC-CD45 (BioLegend, Cat#304038, San Diego, CA, USA), FITC-CD34 (BioLegend, Cat#343504, San Diego, CA, USA), FITC-CD73 (BioLegend, Cat#344016, San Diego, CA, USA), FITC-CD90 (BioLegend, Cat#328108, San Diego, CA, USA), FITC-CD105 (BioLegend, Cat#323204, San Diego, CA, USA), Pacific Blue-conjugated CD235 (BioLegend, Cat#306612, San Diego, CA, USA). Processed cells were sorted on a Sony iCyt SY3200 cell sorter (San Jose, CA, USA) to separate Lineage-negative (Lin−) and Lineage-positive (Lin+) cell populations. Melanoma CTCs were captured in the Lin-fraction, while FITC-positive immune cells were sorted into the Lin+ fraction [10].

Following FACS, 25–50 × 10^3^ cells from the Lin− (melanoma CTCs) and Lin+ (immune cells) populations were harvested and were subjected to RNA isolation. RNA extraction was executed using a Qiagen MicroRNA Isolation kit (Cat#74004, Germantown, MD, USA) in both Lin− and Lin+ cell fractions. Specifically, the whole Lin− fraction was used for RNA isolation. The number of Lin+ fraction was matched to the Lin− cell population. The cells were spun down at 21,000 rcf for 3 min to harvest cells in the pellet. 75 µL Buffer RLT was added to the cell pellet, followed by vigorous vortexing for 5 s to proper homogenize the samples. The samples were centrifuged at 21,000 rcf to get rid of cell debris. 1 volume of 70% ethanol was added to the lysate and mixed by pipetting. The samples were loaded to an RNAse MinElute spin column and centrifuged at 11,000 rcf. The flow-through was discarded. 700 µL Buffer RW1 was added to the spin column, followed by centrifuging at 11,000 rcf for 30 s and discarding the flow-through. 500 µL Buffer RPE was added to the spin column. The samples were centrifuged at 11,000 rcf for 30 s. The flow-through was discarded. 500 µL of 80 ethanol was added to the spin column. The samples were centrifuged at 11,000 rcf for 2 min. The collection tube was discarded. The spin column was placed into a new collection tube and spun at 21,000 rcf for 5 min with an open spin column to dry the membrane. The spin column was placed in a new 1.5 ml (about 0.05 oz) collection tube. 14 µL RNase-free water was added to the center of the spin column. The samples were centrifuged at 21,000 rcf for 1 min to elute the RNA. RNA was stored at − 80 °C.

RNA analysis (Additional file 2), cDNA amplifications and library preparation were performed using the human microarray platform (SMARTer Universal Low Input RNA kit for sequencing from Clontech, Cat#634946, San Jose, CA, USA). For fragmented RNA, the Ion Plus Fragment Library kit (Thermo Fisher, Waltham, MA, USA, Cat#4471252) was used, as reported previously [11–13]. The Ion Proton S5/XL platform (Thermo Fisher, Waltham, MA, USA) was used to sequence the data in the Analytical and Translational Genomics Shared Resource Core at the University of New Mexico [10].

### Identification of tissue-specific genes

We identified tissue-specific genes using the following procedures. Gene expression fold changes were computed between any pair of tissue types. Several thresholds of fold changes and tissue uniqueness were tested. A gene is considered tissue-specific if a minimum threefold increase is observed between the target tissue type and *k*-1 other tissue types individually, where *k* is the total number of tissue types considered. Using this method, a total of 6558 Tissue Specific Genes (TSGs) were selected as training set for our deep learning model.

### Neural network model

We used a fully connected neural network as our deconvolution model, the goal is to create a model recorded as $\widehat{T}= G(X)$ that can automatically analyze the mixture of genetic materials recorded as $X= ({x}_{1},{x}_{2},\ldots ,{x}_{m}),$ and predict the fractions of different target tissue types recorded as $\widehat{T}= ({t}_{1},{t}_{2},\ldots ,{t}_{k})$, where the input $X \in {\mathbb{R}}^{1\times m}$represents all expression data of *m* genes for a sample, the output $\widehat{T}\in {\mathbb{R}}^{1\times k}$ represents all predicted fractions of *k* tissues for a sample, in which, the number of tissues is set to 15 for this study. The higher fraction in $\widehat{T}$ for a particular tissue type can indicate a greater likelihood of cancer development within that same tissue type.

The genetic material can be low in plasma. Thus, missing data is a common scenario in ctcRNA studies. To mask input genes with missing expression values, we disabled the input nodes in our deep-learning model by using the RELU activation function that can simply set missing expression values to 0 resulting in 0 output from the nodes.

$$RELU({x}_{i})= \left\{\begin{array}{ll}0 & for\; {x}_{\text{i}}=NA \\ {x}_{i} & for\; {x}_{\text{i}} \ne NA\end{array}\right.$$

where ${x}_{i}$ represents the expression value of *i*-th gene for a sample.

For the hidden layer with 256 nodes, a RELU function is also used as the layer activation function. For the output layer, a SoftMax function is used as the layer activation function. The SoftMax function is designed to map the output from the hidden layer to a probability distribution with length equal to the number of tissues.

$${t}_{l} =\frac{{e}^{{{t}_{l}}^{\prime}}}{{\sum }_{l=1}^{k}{e}^{{{t}_{l}}^{\prime}}}\quad for\; l=1,\ldots ,k$$

where ${t}_{l}$ represents the probability value of *l*-th tissue. The ${{t}_{l}}^{\prime}$ represents the original data of *l*-th tissue before SoftMax function processing.

The loss function, also called a cost function, is a function that measures the difference between the predicted output value and the actual values. MSE is commonly used as a loss function in regression problems where the goal is to predict a continuous output variable. In this study, we have used MSE as a loss function to evaluate the performance of the model in predicting tissue fractions.

$$MSE(T, \widehat{T})=\frac{1}{n} \sum_{j=1}^{n}{\left({T}_{j}- {\widehat{T}}_{j}\right)}^{2}$$

where ${T}_{j}$ represents the observed probability distribution vector for *j*-th sample and *n* is the size of training data.

Python (version 3.8.3) libraries Keras 2.12.0 and TensorFlow 2.12.0 are used to build this model. The model is trained using the following parameters: batch size is 64, epoch is 200, optimizer strategy is ADAM, the selected GTEx dataset is further processed and standardized using the MinMaxScaler module from the Scikit-Learn library. A training companion function is used to monitor the training process, the monitored variable is validation loss. The original training dataset is further divided into the training set and the validation set. The validation split is 0.1, the validation loss threshold is set to be ${10}^{-5}$, the validation patience is set to 5. This means if the validation loss stopped decreasing for at least ${10}^{5}$ for 5 consecutive epochs, the training of the model stops automatically. The measure is adopted to prevent model overfitting during the training process. The training is done on a Dell workstation T7820 with an Intel(R) Xeon(R) Gold 5220R CPU, 64GB DDR4 RAM and dual Nvidia T1000 graphics cards. The training is also assisted by GPU acceleration, the Nvidia driver version is 528.95, the Nvidia CUDA library version is 11.8, the Nvidia CUDNN library version is 8.9. Furthermore, we also developed a Non-negative Least Squares (NNLS) model to establish a tissue deconvolution performance baseline for comparison with our deep learning model. The NNLS model is built using the R package NNLS version 1.4 with R version 4.2.3.

### Semi in silico data generation

Semi in silico datasets were generated from GTEx and our independent RNA-seq datasets as validation datasets. The semi in silico datasets each contains 1000 samples of mixture tissues. The proportion of the tissues are randomized for each sample. The final semi in silico RNA-seq data for each sample is generated using the following formula:

$$\overrightarrow{{S}_{0}}= \sum _{l=0}^{k}{N}_{l}\times \overrightarrow{{t}_{i}}$$

$\overrightarrow{{S}_{0}}$ is a semi-silico sample gene expression vector containing 6558 TSGs, ${N}_{l}$ a randomly generated ratio with the condition $\sum _{l=0}^{k}{N}_{l}=1$, $\overrightarrow{{t}_{i}}$ is a sample expression vector, specifically for tissue type *l*. For GTEx based semi in silico dataset, we ensured that each sample is comprised of tissue samples from the same donor.

## Results

### Study design

**Fig. 1:**
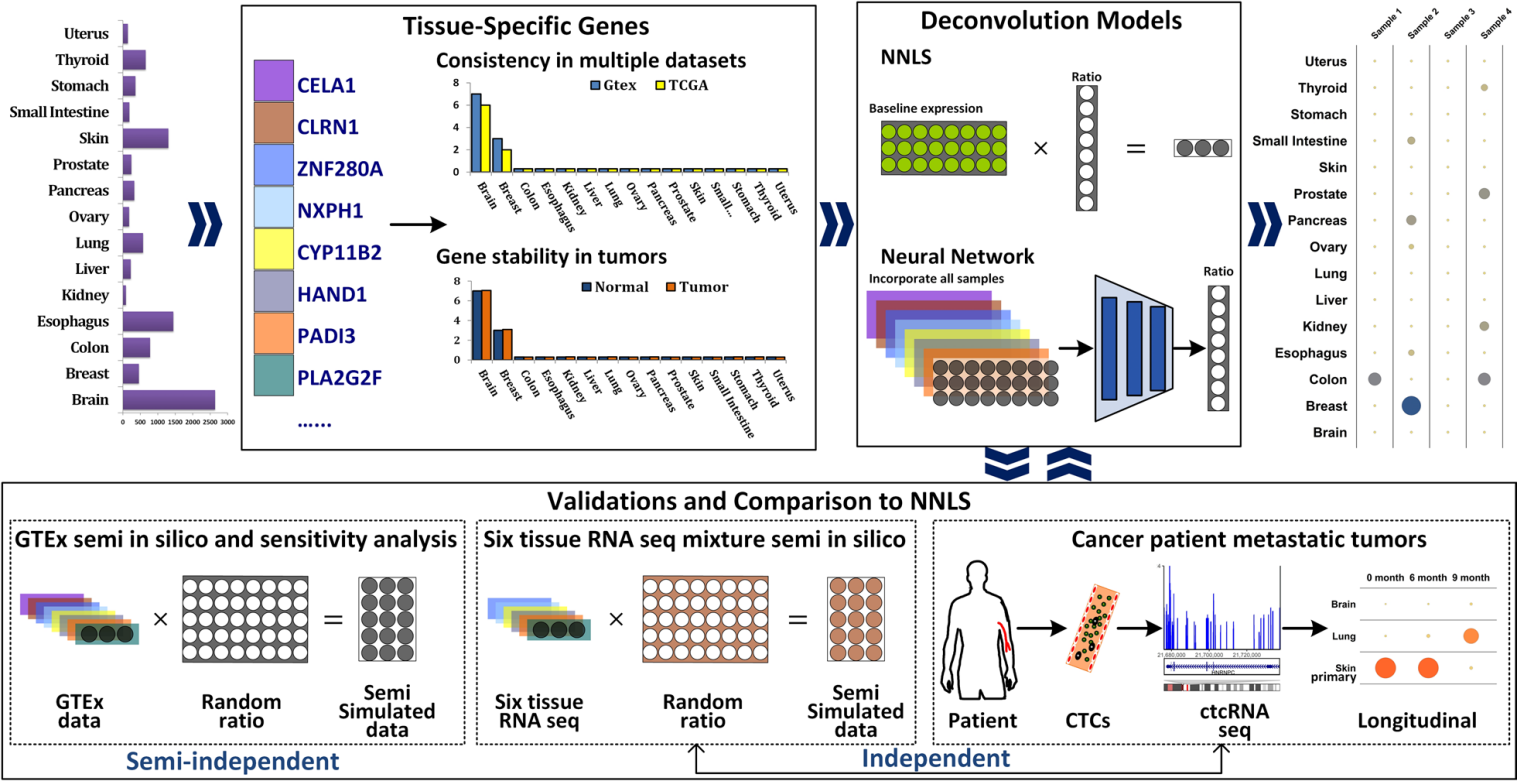
The overall study designs. The neural network deep learning model was trained using GTEx RNA-seq data from TSGs identified in GTEx and TCGA normal tissue. The performance of the model was compared to the traditional NNLS method in three independent validation datasets: (1) Semi in silico dataset from GTEx; (2) Semi in silico dataset from six normal tissue RNA-seq; (3) ctcRNA-seq from a melanoma patient with metastatic tumors

Our study was designed to incorporate extensive consortium data, in-house normal tissue mixture RNA-seq data, and ctcRNA-seq data from a representative cancer patient. The comprehensive study design and conceptual framework are illustrated in Fig. 1. To ensure the robustness of our findings, three distinct validation procedures were undertaken. The first validation involved a semi in silico approach, while the second validation utilized independent RNA-seq analysis of tissue mixtures comprising six tissue types. Finally, a conclusive independent validation was performed using longitudinal ctcRNA-seq data obtained from a patient with metastatic tumors. This final validation exemplified the potential clinical application of early detection of metastatic tumors and highlighting the translational significance of our study. During the validation process, comprehensive comparisons were conducted between the deep learning approach and the NNLS method, highlighting the consistent advantages exhibited by the deep learning approach across various scenarios.

### Tissue-specific genes (TSG)

**Fig. 2:**
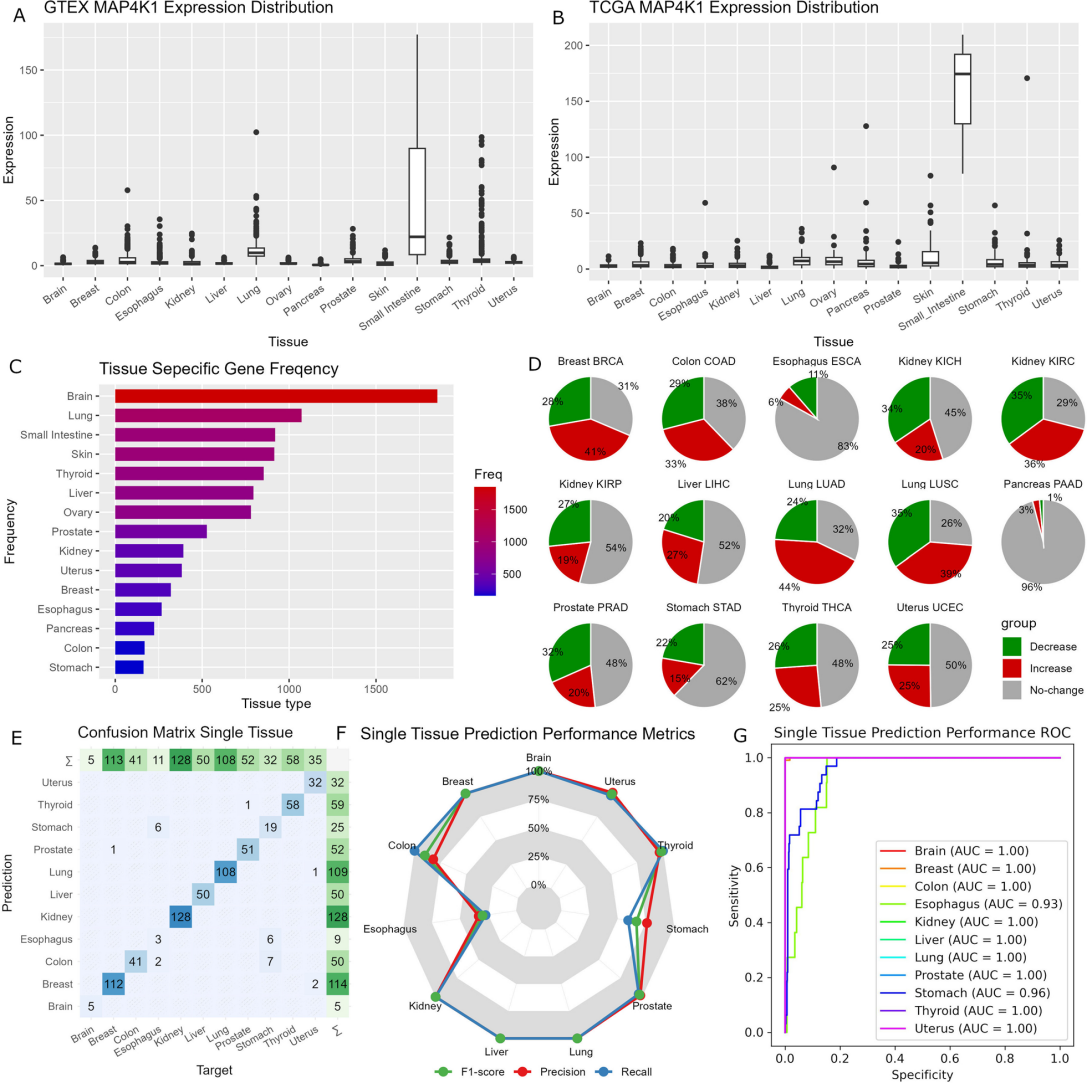
Illustration of our TSG analyses. **A** Boxplots show that the gene MAP4K1 is primarily expressed only in the small intestine in GTEx. **B** Boxplots show that the gene MAP4K1 is primarily expressed only in the small intestine in normal tissues in TCGA which confirms the finding in GTEx. **C** A Bar chart that depicts the number of TSGs in each tissue type. Large variations in the number of TSGs can be observed. **D** Pie charts that the results from gene stability tests. Gray color indicates the proportion of TSGs that showed no notable change between tumor and normal. Red color indicates the proportion of TSGs that showed upregulation in tumors. Green indicates the proportion of TSGs that showed downregulation in tumors. **E**–**G** shows results from independent validation of the single tissue deep learning prediction model using TCGA normal tissue RNA-seq data. **E** Confusion matrix that shows overall high accuracy of the single tissue deep learning prediction model. **F** A polygon plot that demonstrates performance of the single tissue deep learning prediction model in F1 score, precision, and recall. **G** ROC curves show the high performance of the single tissue deep-learning prediction model

We selected 15 tissue types (brain, breast, colon, esophagus, kidney, liver, lung, ovary, pancreas, prostate, skin, small intestine, stomach, thyroid, and uterus). These tissue types were chosen based on the availability of readily accessible RNA-seq data and their prevalence as common cancer sites. To identify tissue-specific genes (TSGs), we initially utilized the GTEx RNA-seq dataset. Subsequently, we validated these TSGs with TCGA’s normal tissue RNA-seq dataset. For instance, the TSG MAP4K1 was identified exclusively in the small intestine within the GTEx RNA-seq data and further confirmed through verification in the TCGA normal tissue RNA-seq data (Fig. 2A, B). The validation process serves to ensure that the observed tissue-specificity is not influenced by noise or batch effects originating from a single dataset. Ultimately, our tissue-specificity criteria led to the identification of a total of 6,558 genes that met the established criteria. Among the tissue types considered, the average number of TSG per tissue was 642. The brain exhibited the highest number of tissue-specific genes (TSGs), totaling 1853, whereas the stomach presented the lowest number of TSGs, amounting to only 163 (Fig. 2C).

Next, we conducted a TSG stability test within tumor samples, aiming to demonstrate that a considerable proportion of the identified tissue-specific genes (TSGs) remain unaffected by tumorigenesis. To achieve this, we conducted gene expression analyses comparing tumor samples to their corresponding normal tissue counterparts, utilizing the TCGA RNA-seq data (Fig. 2D). However, this analysis was limited to 12 tissue types due to the unavailability of adjacent normal tissue in certain cancer types. On average, approximately 50% of the tissue-specific genes (TSGs) exhibited no significant difference in expression between tumor and normal samples, with a range spanning from 26% in lung cancer to 96% in pancreatic cancer. Conversely, around 25% of the TSGs demonstrated upregulation in tumor samples, ranging from 3% in pancreatic cancer to 44% in lung cancer. Additionally, approximately 25% of the TSGs displayed downregulation in tumors, varying from 1% in pancreatic cancer to 35% in lung cancer. The dysregulation of gene expression during the transition from normal to tumor states can influence the effectiveness of TSGs in tissue deconvolution analyses. However, our subsequent analysis reveals that deep learning models exhibit greater resilience towards variations in gene expression, mitigating the impact of these dysregulations.

To establish the feasibility of a deep learning approach, we initially developed a deep learning model specifically tailored for single tissue prediction, utilizing the GTEx dataset. As anticipated, this model demonstrated exceptional performance when evaluated with the TCGA normal tissue RNA-seq data as an independent testing dataset. The overall accuracy achieved reached 96% (Fig. 2E). Moreover, the F1 score, recall, and precision metrics of this model also exhibited significant levels of performance (Fig. 2F). Receiver operating characteristic (ROC) curves further substantiated these findings, as most tissue types displayed an area under the curve (AUC) value close to one, indicating high discriminatory power (Fig. 2G).

### Validation by semi in silico data

To evaluate and compare the performance of our deep-learning tissue deconvolution model, we conducted three distinct validation tests and comparative analyses. The initial validation approach involved utilizing a semi in silico dataset generated from the RNA-seq data obtained from the Genotype-Tissue Expression (GTEx) project. The GTEx dataset is particularly suitable for semi in silico RNA mixtures derived from multiple tissues, as it encompasses multiple tissue samples from individual donors. We constructed a semi in silico dataset comprising 1000 randomized tissue mixture RNA-seq samples using the methodologies outlined in the Methods section. This allowed us to simulate diverse RNA mixtures and create a comprehensive dataset for rigorous evaluation and validation of our deep-learning model.

**Fig. 3:**
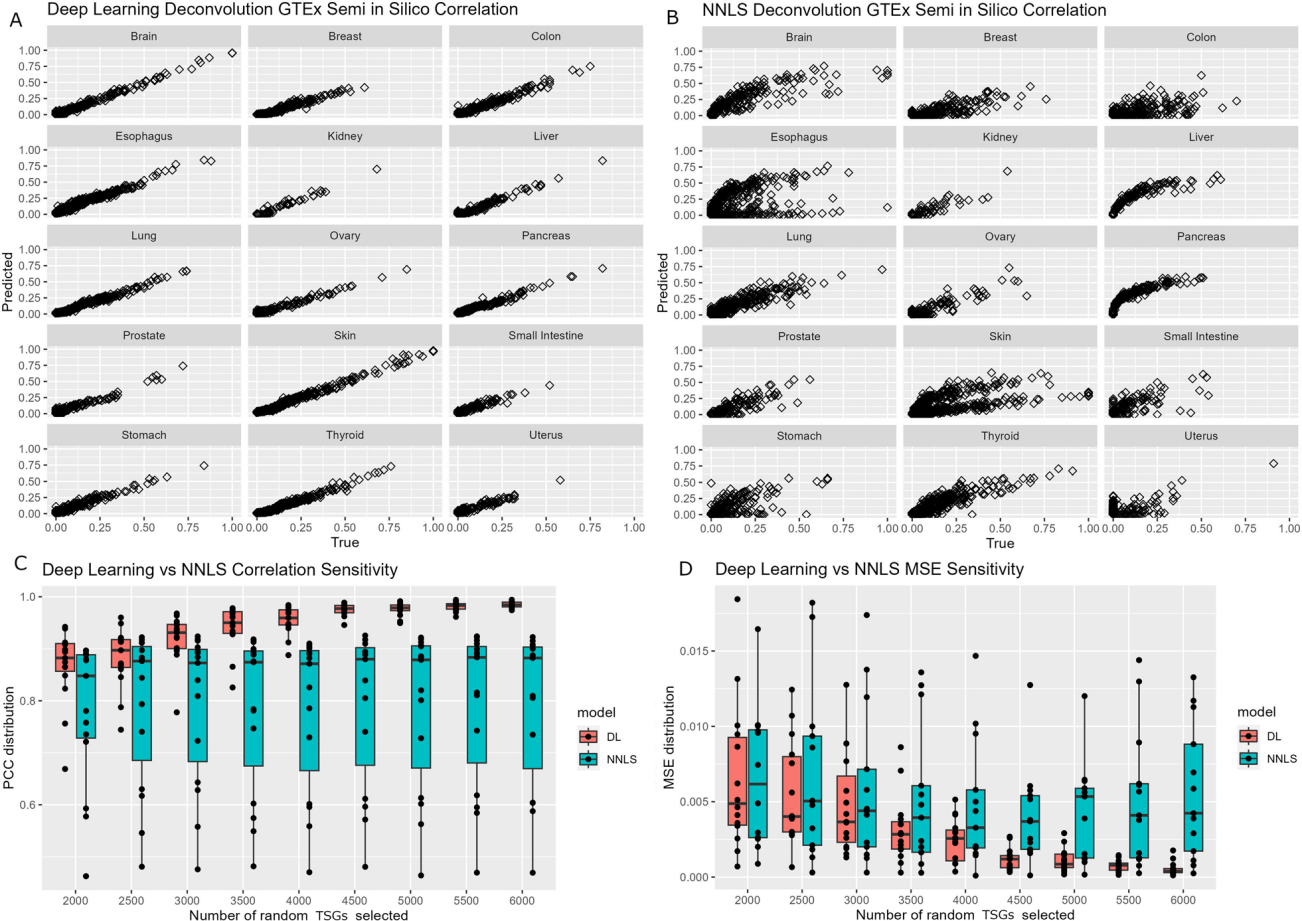
Data analyses illustrating validation using semi in silico GTEx data and comparison between deep learning model and NNLS method. **A** Scatter plots that depict the correlation between randomized tissue contribution and predicted tissue contribution by deep learning model. **B** Scatter plots that depict the correlation (*r*) between randomized tissue contribution and predicted tissue contribution by the NNLS method. **C** Boxplots that show higher performance in the sensitivity analysis by deep-learning model. **D** Boxplots that show smaller MSE in the sensitivity analysis by deep-learning model

The identical semi in silico dataset was subjected to deconvolution using both the deep learning model and the NNLS method. The efficacy of these methods was assessed by employing two performance metrics: Pearson’s correlation coefficient and mean squared error (MSE). The calculation of correlation (*r*) was computed between the randomly generated tissue contributions and the predicted tissue contributions across the 1000 semi in silico tissue mixture samples. On average, deep-learning model achieved a correlation of 0.98 and exhibited a small variation (Fig. 3A), while NNLS had an average correlation of 0.79 (Range: 0.47 in colon to 0.97 in ovary) (Fig. 3B). MSE was calculated as the mean distance from each randomized tissue contribution of each semi in silico sample to the predicted regression model. Additionally, a sensitivity analysis was conducted to assess the impact of missing data on the two methods. Random selections of genes, ranging from 2000 to 6000 tissue-specific genes (TSGs) with 500 incremental intervals, were used to evaluate both the deep learning model and the NNLS approach. Notably, the deep learning model consistently outperformed the NNLS method in terms of correlation (Fig. 3C) and MSE (Fig. 3D). This observation underscores the deep-learning model’s resilience to the challenges posed by missing data, which are prevalent in real-world applications.

### Validation by normal tissue mixture RNA-seq

The previous validation utilizing the semi in silico GTEx data demonstrated promising outcomes for the deep-learning model. Nonetheless, it is worth noting that despite the randomization employed, the GTEx dataset was utilized for both training and validation, thereby lacking complete independence and potentially introducing bias. To address this concern, we conducted RNA-seq experiments on six distinct normal tissues (brain, breast, colon, kidney, liver, and lung) sourced from the Human Tissue Repository at the University of New Mexico. This approach ensured absolute independence from the GTEx dataset, bolstering the reliability and unbiased nature of our validation procedure.

Employing the aforementioned strategy, we constructed a semi in silico dataset comprising 1000 samples using the RNA-seq data obtained from the six normal tissues. This RNA-seq data contained 4973 of the 6558 TSGs found previously, serving as a tangible demonstration of the potential disparities that real world application may encounter.

**Fig. 4:**
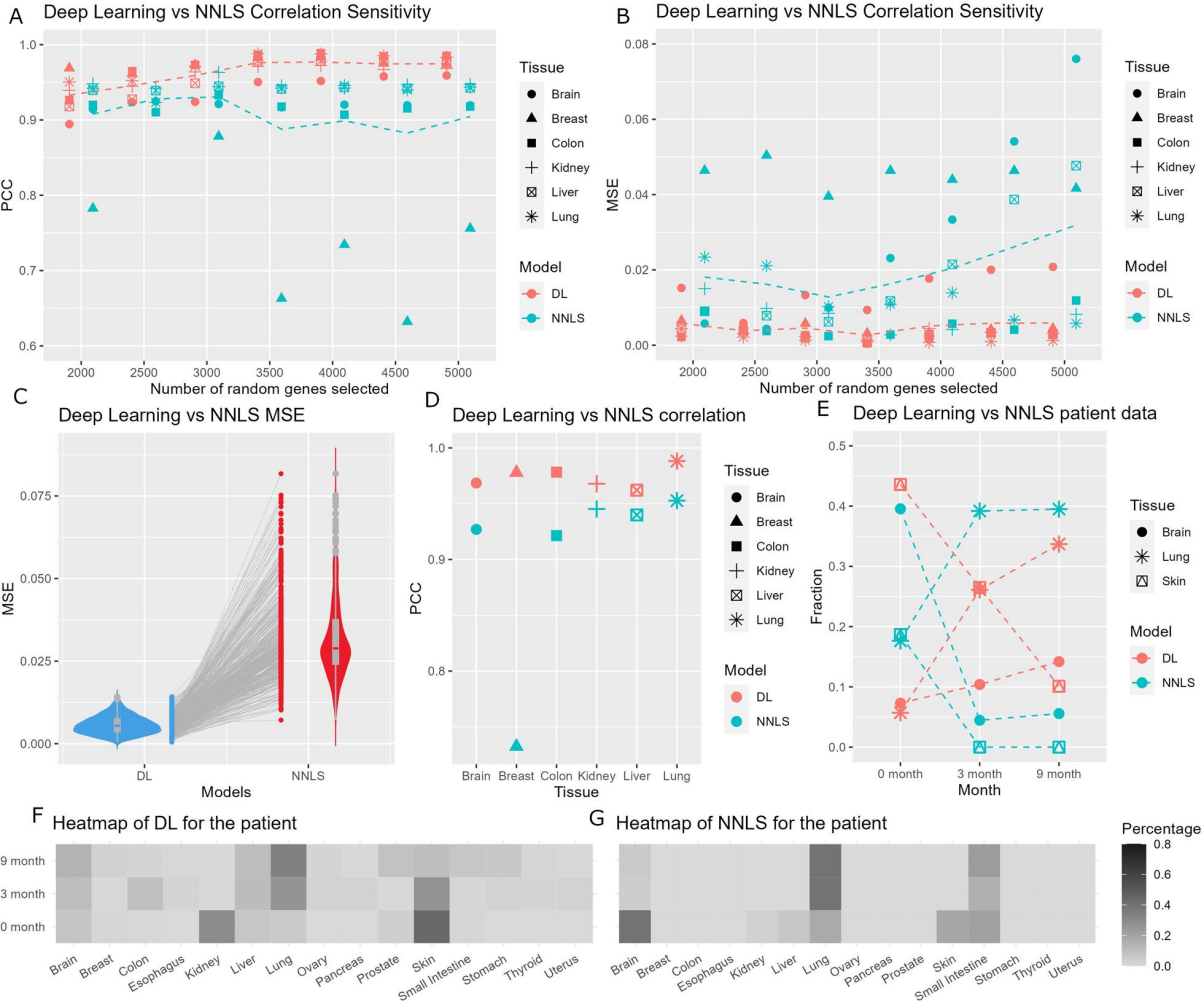
The results from validation by normal tissue RNA-seq data and patient ctcRNA-seq data. **A** A dot plot that shows higher correlation (*r*) achieved by deep learning model in the sensitivity analysis. **B** A dot plot that shows small MSE achieved by deep learning model in the sensitivity analysis. For A and B, the dotted line indicates the mean value. **C** A box violin and dot combination plot that shows smaller MSE for all 1000 semi in silico samples. **D** A dot plot that shows overall higher correlation by deep learning model. **E** A dot plot that shows the proportion of tissue contribution in 9 month follow up for a melanoma patient with brain and lung metastatic tumors. **F** A heatmap that depicts the complete 15 tissue results by deep learning model. **G** A heatmap that depicts the complete 15 tissue results by the NNLS method

The performance of tissue deconvolution was assessed through the utilization of both deep learning and NNLS methods, employing Pearson’s correlation and MSE as performance metrics. Our deep learning model demonstrated outstanding proficiency by achieving an average correlation of 0.97 (with a range of 0.95 in brain tissue to 0.98 in lung tissue). In contrast, the NNLS method yielded an average correlation of 0.89 (with a range of 0.71 in breast tissue to 0.95 in kidney tissue). Sensitivity analyses were conducted to scrutinize the performance of the two methods at various with randomized gene sets from 2000 TSGs to 6000 TSGs at 500 incremental intervals. The results showed that the deep learning model outperformed the NNLS measured by both correlation (Fig. 4A) and MSE (Fig. 4B). The evaluation of 1000 semi in silico samples demonstrated consistently lower MSE values for the deep learning model compared to the NNLS method (Fig. 4C). The overall correlation results also favored deep learning (Fig. 4D). These compelling findings provide further substantiation of the advantageous capabilities of deep learning in the domain of tissue deconvolution.

### Tissue deconvolution using ctcRNA

We analyzed a longitudinal ctcRNA-seq dataset obtained from a melanoma patient presenting with metastatic tumors in the lung and brain. The ctcRNAs were collected at three distinct time points: 0, 3, and 9 months following the initial diagnosis. Utilizing both the deep learning and NNLS methods, we endeavored to discern the tissue origin of the ctcRNAs (Fig. 4E). Upon analysis, we observed that both methods successfully detected the presence of skin tissue at the 0-month time point. However, the deep-learning approach exhibited a higher proportion of skin detection compared to the NNLS method. Notably, the NNLS method failed to identify any skin tissue at the 3 and 9-month time points. Furthermore, as time progressed, the deep-learning model discerned an increasing proportion of lung and brain tissues, indicating the progression of metastasis. In contrast, the NNLS method only identified an increased proportion of lung tissue but did not detect any changes in the brain tissue proportion.

The comprehensive outcomes encompassing all 15 tissues are presented in Fig. 4F for deep learning and Fig. 4G for NNLS. Notably, both methods consistently detected negligible or nearly absent content for female organs such as breast, ovary, and uterus, which aligns with the patient’s male gender. It is important to note that unlike the semi in silico datasets, the precise ground truth for patient samples remains unknown. Therefore, drawing definitive conclusions when comparing deep learning and NNLS methods proves to be challenging due to the complexity and the inherent limitations of the dataset. Nonetheless, the observed results remained in concordance with the overall trend deduced from the patient’s metastatic history and gender. This agreement between the findings and the known clinical information further accentuates the potential clinical utility of the employed techniques.

## Discussion

Deep learning has shown significant promise in various areas of cancer research due to its ability to handle complex data, provide accurate predictions, integrate multiple data modalities, and facilitate knowledge discovery which makes it a valuable tool in cancer research. It has the potential to transform cancer diagnosis, treatment, and precision medicine, ultimately improving patient care and outcomes in the fight against cancer [14]. For example, deep learning has been used in cancer diagnosis [15], drug discovery [16], prognosis prediction [17], and cancer imaging [18].

In the present study, we employed neural network deep learning for tissue deconvolution. Previous research has demonstrated the feasibility of tissue deconvolution using traditional statistical regression methods such as non-negative least squares (NNLS) applied to cfDNA or cfRNA [3, 4]. However, these approaches rely on predefined tumor TSG panels and may not fully capture the inherent data variation during model training. Conversely, deep-learning techniques can leverage the entirety of the training dataset, enabling the model to learn and capture the inherent variation between samples. Considering this advantage, we hypothesized that a deep learning-based tissue deconvolution model might outperform the traditional NNLS approach in terms of accuracy and performance.

The findings of our study revealed two significant advantages associated with the implementation of a deep-learning model. Firstly, the deep-learning model exhibited superior performance in both validation datasets compared to the alternative approach. This enhanced performance can be attributed to the model’s capability to effectively capture the inherent variation within the data. Secondly, the deep-learning model demonstrated reduced susceptibility to the challenges posed by missing data, which are inevitable in real-world applications. This advantage can be attributed to the flexible structure inherent in neural networks, allowing them to handle missing data more effectively. We have demonstrated that deep-learning models do not consistently outperform traditional machine learning algorithms in all scenarios [19]. However, in the context of tissue deconvolution, deep-learning emerges as a superior approach primarily due to the complex nature of the data involved in tissue deconvolution and the flexible model structure inherent to deep-learning methodologies.

In addition to cfRNA, we aimed to show that tissue deconvolution can be done with ctcRNAs which hold significant importance in cancer research and clinical applications. As a non-invasive biomarker obtained through liquid biopsies, ctcRNA allows for real-time insights into tumor heterogeneity, dynamic monitoring of disease progression, and early detection of metastasis.

When tumor cells disseminate to distant organs, they can establish new tumors in those sites. These metastatic tumors may exhibit certain characteristics that are specific to the metastatic site. This phenomenon is known as target organ metastatic specificity organ or organotropism [20]. The microenvironment of different organs can influence the behavior and properties of tumor cells, leading to variations in gene expression patterns, cellular morphology, and other characteristics between primary and metastatic tumors. Therefore, metastatic tumors may exhibit properties distinct from the primary tumor and specific to the organ to which they spread.

The utilization of cell-free RNA (cfRNA) or circulating tumor cell RNA (ctcRNA) enables the possibility of tissue deconvolution, primarily due to the presence of tissue-specific genes (TSGs). Nevertheless, it is essential to recognize that TSGs may also impose limitations. When dealing with tissues lacking TSGs or possessing a limited number of them, distinguishing RNAs originating from these tissues becomes challenging as they cannot be readily distinguished from other sources. In the context of our deep learning model, we assume that the sum of contributions from the 15 tissue types amounts to one. However, in real-world scenarios, cfRNA or ctcRNA may originate from more than 15 tissue types, further complicating the deconvolution process. In a previous study focused on tissue deconvolution [4], a linear regression model was employed to derive the contribution of each tissue type included in the investigation. This model aimed to establish a linear correlation between sample expressions and expression median values. The model can be denoted as $y=X\beta + \epsilon$, where $y$ is the sample expression, $X$ is the median expression matrix for different tissue types for all genes. $\beta$ is the vector of tissues’ contribution. ${min}_{\beta }\left({\left\|X\beta -y\right\|}^{2}\right)$ is used to calculate the $\beta$ vector, where all ${\beta }_{i}$ ≥ 0, $\sum {\beta }_{i}\le 1$. This function is solved by quadratic programming which is remarkably similar to the NNLS algorithm. It is noteworthy that the summation of tissue contributions is permitted to be less than 1 to accommodate the presence of noises originating from tissue types not explicitly included in the model. To address this scenario, it is possible to incorporate a noise output node into the output layer of the deep learning model. A predetermined noise level must be assigned to this node, which can be an arbitrary value or estimated based on prior experience. The deep learning model would function exactly except for the summation of the contribution. Through our analysis of longitudinal ctcRNA-seq data obtained from a melanoma patient, we observed outcomes that closely mirrored the patient’s metastatic history and gender. This finding highlights the relevance and accuracy of our approach in capturing and reflecting the patient-specific characteristics of the disease progression and biological features.

In summary, our results highlight the benefits of utilizing a deep learning model in tissue deconvolution, including its improved performance in the validation datasets and its resilience to missing data issues. Furthermore, we have demonstrated a novel application of ctcRNA for tissue deconvolution and provided evidence supporting its feasibility reliability. The incorporation of deep-learning models with ctcRNA has broad clinical implications for early detection of metastasis, treatment decision-making, disease monitoring, prediction of treatment resistance, and biomarker discovery, amongst others. The analysis of ctcRNA with deep learning holds great promise for advancing our understanding of cancer biology and enhancing patient care.

### Supplementary Information

**Additional file 1**: RNA-seq data from normal tissues.

**Additional file 2**: ctcRNA-seq data from the patient with metastasis tumors.

## Data Availability

All codes used in this study are available as Additional data published. All codes with updated versions, if applicable, can be accessed at the following GitHub link: https://github.com/yay135/GTEx_NNLS_Deep_Learning. Previously, ctcRNA-Seq method has been incorporated in a US patent application (STC. UNM Ref. No. 2023-036-01). The RNA-seq data generated for this project is available as Additional files 1, 2.

## Declarations

### Concent for publication

All authors give their consent to publish this manuscript.
